# Using feature optimization and LightGBM algorithm to predict the clinical pregnancy outcomes after *in vitro* fertilization

**DOI:** 10.3389/fendo.2023.1305473

**Published:** 2023-11-29

**Authors:** Lu Li, Xiangrong Cui, Jian Yang, Xueqing Wu, Gang Zhao

**Affiliations:** ^1^ School of Basic Medicine, Anhui Medical University, Hefei, China; ^2^ Center of Reproductive Medicine, Children’s Hospital of Shanxi and Women Health Center of Shanxi, Taiyuan, China; ^3^ School of Information, Shanxi University of Finance and Economics, Taiyuan, China; ^4^ Department of Electronic Science and Technology, University of Science and Technology of China, Hefei, China

**Keywords:** IVF, clinical pregnancy, LightGBM, machine learning, prediction models

## Abstract

**Background:**

According to a recent report by the WHO, approximately 17.5\% (about one-sixth) of the global adult population is affected by infertility. Consequently, researchers worldwide have proposed various machine learning models to improve the prediction of clinical pregnancy outcomes during IVF cycles. The objective of this study is to develop a machine learning(ML) model that predicts the outcomes of pregnancies following *in vitro* fertilization (IVF) and assists in clinical treatment.

**Methods:**

This study conducted a retrospective analysis on provincial reproductive centers in China from March 2020 to March 2021, utilizing 13 selected features. The algorithms used included XGBoost, LightGBM, KNN, Naïve Bayes, Random Forest, and Decision Tree. The results were evaluated using performance metrics such as precision, recall, F1-score, accuracy and AUC, employing five-fold cross-validation repeated five times.

**Results:**

Among the models, LightGBM achieved the best performance, with an accuracy of 92.31%, recall of 87.80%, F1-score of 90.00\%, and an AUC of 90.41%. The model identified the estrogen concentration at the HCG injection(etwo), endometrium thickness (mm) on HCG day(EM TNK), years of infertility(Years), and body mass index(BMI) as the most important features.

**Conclusion:**

This study successfully demonstrates the LightGBM model has the best predictive effect on pregnancy outcomes during IVF cycles. Additionally, etwo was found to be the most significant predictor for successful IVF compared to other variables. This machine learning approach has the potential to assist fertility specialists in providing counseling and adjusting treatment strategies for patients.

## 1 Introduction

The World Health Organization defines infertility as the situation where a married couple is unable to achieve pregnancy after one year or more of regular, unprotected sexual intercourse (1). Infertility is a global life crisis that impacts individuals worldwide (2). According to reports, the prevalence of infertility among couples of reproductive age worldwide is estimated to range from 10-25%, affecting an estimated 48 to 180 million couples (3). Since the inception of *in vitro* fertilization (IVF) - the groundbreaking assisted reproductive technology (ART) procedure with the birth of the world’s first human baby in the UK in 1978, over nine million IVF babies have been born worldwide (4). Globally, the number of children conceived using ART surpasses 8 million, comprising 2-6% of the total birth population across European nations and 1.743% of China’s total birth population (5–7). ART techniques primarily comprise controlled ovarian hyperstimulation (COH), *in vitro* fertilization and embryo transfer (IVF-ET), intracytoplasmic sperm injection (ICSI), preimplantation genetic diagnosis (PGD), frozen embryo transfer (FET), and *in vitro* maturation (IVM) of oocytes. Although advances in clinical and laboratory techniques over the past decades have substantially improved pregnancy rates in assisted reproductive technology (ART) (8), the live birth rate per cycle remains below 29.1% (9). Following several cycles, a significant number of patients encounter failure, with a remaining rate of 38% to 49% of couples not attaining success (10). The treatment process imposes various burdens on patients, including mortality, adverse drug reactions, psychological distress, social challenges, and economic difficulties. Additionally, ART encompasses multiple intricate stages that require significant time and financial investment (11, 12). Therefore, it is imperative for infertile couples to possess a thorough comprehension of the potential success rate of ART. Moreover, they should meticulously consider the consequent risks, including 42 financial and physical implications, prior to determining whether ART is a viable option for procreation (10, 11).

Machine learning is an artificial intelligence (AI) technology that utilizes data analysis to enable computers to learn patterns and models. This empowers computers with the capacity to make independent decisions and predictions. Particularly in clinical prediction, machine learning plays a significant role owing to its strong decision-making capability and its proficiency in analyzing high-dimensional data (13–15). Machine learning, an advanced approach to computer modeling, has the potential to greatly enhance predictive capability when compared to traditional methods. It can take into account variable interactionsand continuously integrate new data to update algorithms (16). Machine learning has demonstrated promising applications and potential in the field of reproductive medicine, specifically in the domains of embryo grading and predicting embryo implantation rates (17). Multiple studies have investigated the use of machine learning to predict success rates in *in vitro* fertilization (IVF). These studies have identified several factors that impact the success rate, including age (18–20), causes of infertility (21), embryo quality (22), and dosage of follicle stimulating hormone (FSH) (19), among others. It is worth noting that the extent to which these factors influence each patient can vary across different IVF cycles. As a result, accurately predicting the outcomes of assisted reproductive technology (ART) poses a considerable challenge for future research on machine learning in this domain (23).

This study employs individual characteristics, clinical indicators, and laboratory indicators as variables to predict the clinical pregnancy rate following embryo implantation through the use of machine learning techniques. It assesses and compares the predictive capabilities of six classification algorithms with regards to the success rate.

## 2 Materials and methods

### 2.1 Patients

IVF patients who underwent fresh embryo transfers at Children’s Hospital of Shanxi and Shanxi Women’s Health Center between March 2020 and March 2021 were enrolled in our study. Exclusion criteria included patients with (1) oocyte donation cycles, (2) cryopreserved and warmed oocytes, and (3) combined cryopreserved and warmed embryo transfers. A total of 840 patients were included as the training set for building our model.

### 2.2 Controlled ovarian hyperstimulation, embryo culture, and pregnancy ascertain

The patient received ovulation induction treatment. Follicle growth and development were monitored using B-ultrasound. When the follicles reached a diameter greater than 18 mm, the patient received a muscle injection of 10,000 IU of human chorionic gonadotropin. After 36 hours, the follicle aspiration was performed via transvaginal puncture under B-ultrasound guidance. The retrieved eggs were fertilized through IVF or ICSI procedures in the laboratory. On the 3rd to 5th day after fertilization, high-quality embryos were selected for transfer. Luteal phase support was subsequently provided. Biochemical pregnancy was confirmed 10 days after the transfer by measuring HCG levels in the blood. Approximately 30 days after the transfer, a gestational sac indicative of clinical pregnancy was observed through B-ultrasound.

### 2.3 Model construction and feature selection

**Figure 1:**
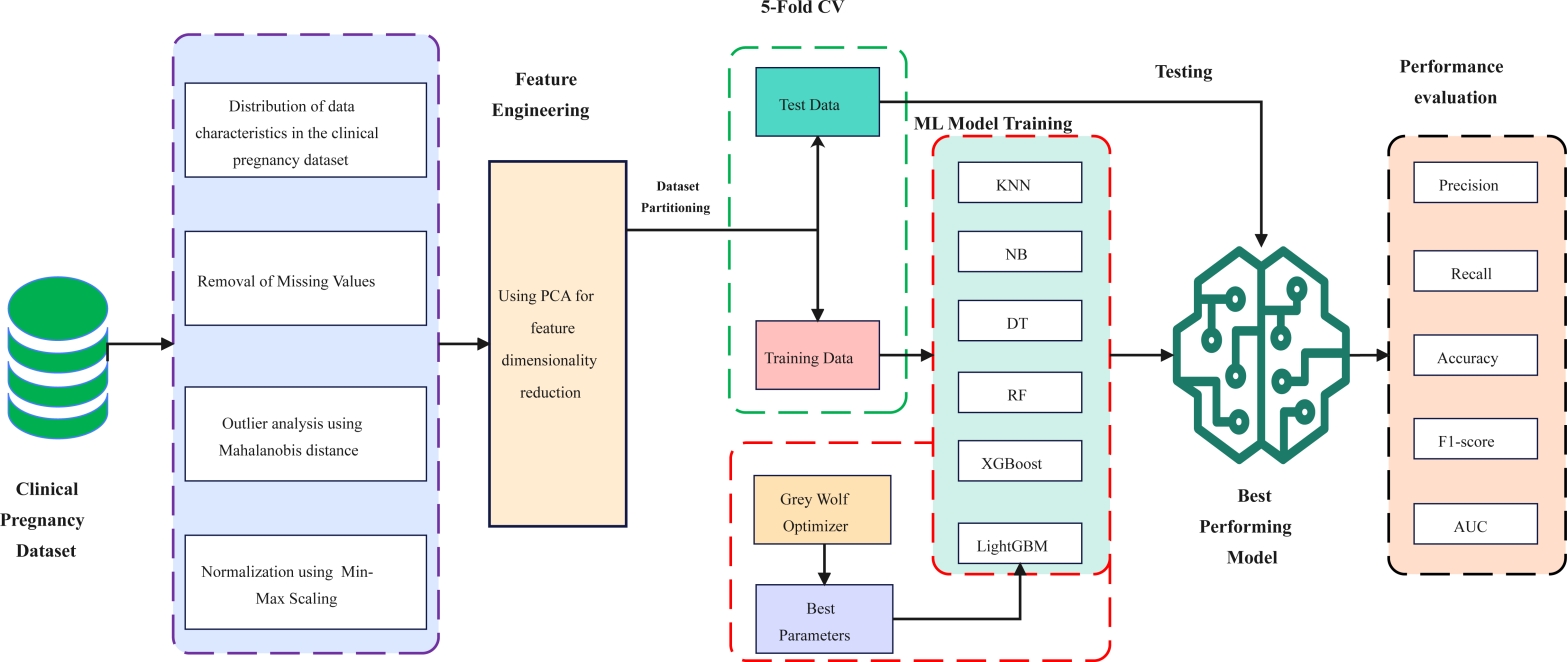
The process of the proposed framework.

**Figure 1** shows the machine learning framework for predicting clinical pregnancy outcomes after *in vitro* fertilization.

#### 2.3.1 Data pre-processing

In data analysis in the medical field, having a good awareness of data is crucial because the quality of the data will directly affect the predictive performance of machine learning models. In this article, we use statistical parameters (i.e., median) to impute missing values for corresponding attributes and utilize Mahalanobis Distance(MD) for outlier detection. Additionally, to ensure that clinical features in the test dataset are treated equally across different scales, we employ the min-max scaling method which guarantees their equal contribution to model fitting.


(1) $$D_{Scaled}=\frac{D-D_{min}\left(axis=0\right)}{D\_max\left(axis=0\right)-D_{min}\left(axis=0\right)}$$

Here D represents the instances to be scaled. D*max*(*axis* = 0) represents the maximum values of feature vectors in the training instances, while D*min*(*axis* = 0) represents the minimum values of feature vectors in the training instances.

#### 2.3.2 Feature selection

Principal Component Analysis(PCA) is a commonly used data dimensionality reduction algorithm, which aims to reduce the dimensions of a dataset and extract the most important features. Its main objective is to transform the original high-dimensional features into new orthogonal features while preserving as much important information from the original data as possible. The matrix *Z* is composed of data collected.


(2) $$\text{Z}=\left(\begin{array}{cccc} z_{11} & z_{12} & \cdots & z_{1m}\\ \vdots & \vdots & \vdots \\ z_{n1} & z_{n2} & \cdots & z_{nm} \end{array}\right)$$

- Calculate the covariance matrix between features, denoted as *C* = (*ZTZ*)*_m×m_*. The covariance matrix is a *m_×_m* symmetric matrix that relates the covariances and variances of multiple variables. We can decompose any matrix into three distinct matrices based on singular value decomposition (SVD).
- Perform an eigenvalue decomposition on the covariance matrix to obtain eigenvalues and eigenvectors.
- Sort the eigenvalues in descending order and select the eigenvectors corresponding to the *k* largest eigenvalues.
- Project the original data onto the subspace formed by selected feature vectors to obtain the reduced-dimensional dataset.

#### 2.3.3 LightGBM

LightGBM (24) is an algorithm based on gradient boosting trees, aiming to enhance prediction accuracy by stacking weak classifiers. Compared to standard gradient boosting tree algorithms, LightGBM uses histogram optimization to segment continuous features, saving memory and speeding up computation. The decision trees are grown using a leafwise strategy and limited depth to prevent overfitting, effectively improving the accuracy and robustness of model predictions. To address overfitting caused by complex trees, regularization terms are introduced in the loss function, which can be represented by the following formula.


(3) $$f_{obj}^{k}=\sum_{i=1}^{n}Loss\left({\hat{y}}_{i}^{k},y_{i}\right)+\sum_{i=1}^{k}\omega \left(f_{i}\right)=\sum_{i=1}^{n}Loss\left({\hat{y}}_{i}^{k-1}+f_{k}\left(x_{i}\right),y_{i}\right)+\sum_{i=1}^{k}\omega \left(f_{i}\right)$$

Where, $y_{i}$ represents the actual value of the label, $\omega \left(f_{i}\right)$ is the regularization term, and ${\hat{y}}_{i}^{k-1}$ is the current computed value of the model. By using a second-order Taylor expansion to expand the objective function, we obtain equation (4).


(4) $$f\left(x+\text{Δ}x\right)=f\left(x\right)+f^{\prime }\left(x\right)\cdot_{\text{Δ}}x+\frac{1}{2}f^{\prime \prime }\left(x\right)\cdot_{\text{Δ}}x^{2}$$

The second-order Taylor expansion of the loss function is shown as follows.


(5) $$\sum_{i=1}^{n}Loss\left({\hat{y}}_{i}^{k-1}+f_{k}\left(x_{i}\right),y_{i}\right)=\sum_{i=1}^{n}\left[\begin{array}{c} Loss\left({\hat{y}}_{i}^{k-1},y_{i}\right)+Los^{\prime }\left({\hat{y}}_{i}^{k-1},y_{i}\right)\cdot f_{k}\left(x_{i}\right)\\ +\frac{1}{2}Loss^{\prime \prime }\left({\hat{y}}_{i}^{k-1},y_{i}\right)\cdot f_{k}^{2}\left(x_{i}\right) \end{array}\right]$$

Replacing the first-order derivative of the Loss function for the *i-th* data with *u*, and replacing the second-order derivative of the Loss function for the *j-th* data with *v*, then the objective function can be simplified as:


(6) $$f_{obj}^{k}=\sum_{i=1}^{n}[Loss\left({\hat{y}}_{i}^{k-1},y_{i}\right)+u_{i}f_{k}\left(x_{i}\right)+\frac{1}{2}\nu_{i}f_{k}^{2}\left(x_{i}\right)]+\sum_{i=1}^{k}\omega \left(f_{i}\right)$$

When using LightGBM in practice, its core parameters such as maximum tree depth, learning rate, and threshold value will affect the accuracy of model recognition. In particular, the selection of the threshold value is crucial for accurate prediction of clinical pregnancy. Therefore, obtaining the optimal parameters for LightGBM is a key step in improving model prediction accuracy.

Gray Wolf Optimization(GWO) (25) is a swarm intelligence optimization algorithm designed based on the simulation of grey wolf hunting behavior. This algorithm has advantages such as simplicity, speed, and ease of implementation. In GWO, the social hierarchy of grey wolf individuals is divided in order of α, β, γ, and δ. Where α represents the highest fitness solution, β, γ, and δ represent the second-best solution, third-best solution, and other solutions respectively. The remaining individuals are directed by the top three best solutions (α, β and γ) to update their positions using the following formula.


(7) $$\left\{ \begin{array}{c} W_{1}\left(t+1\right)=W_{\alpha }\left(t\right)-X_{1}\left|Y_{1}W_{\alpha }\left(t\right)-W\left(t\right)\right|\\ W_{2}\left(t+1\right)=W_{\beta }\left(t\right)-X_{2}\left|Y_{2}W_{\beta }\left(t\right)-W\left(t\right)\right|\\ W_{3}\left(t+1\right)=W_{\gamma }\left(t\right)-X_{3}\left|Y_{3}W_{\gamma }\left(t\right)-W\left(t\right)\right| \end{array}\right.$$

In the standard GWO, X and Y are used as coefficients, where W(t) represents the current position of an individual and W(t+1) represents the updated position of a grey wolf. However, during the initialization phase, the population positions are randomly generated. This random search strategy may cause the initial positions of individuals to concentrate around certain extreme points, leading to missing crucial information and affecting the convergence speed and accuracy of the model. Therefore, in order to ensure that the population is uniformly distributed with diversity during the initialization phase, Halton sequence is adopted here for population initialization. Halton sequence is a type of sequence that can generate uniformly distributed random numbers within a search space with low discrepancy. The following formula can be used to obtain Halton sequence H(n) with base *k*(*k* ≥ 2):


(8) $$s=\sum_{n=0}^{N}r_{n}k^{n}$$

Where, *r_n_* ∈{0,1,…, *k*-1}(*n*=0, 1,…, *N*). Furthermore, we define a basic inverse function, denoted as φ*_k_* (*n*) = (0.a_0_a_1_…a*_M_*)_k_ = a_0_/*k*+…+a_M_/*k* ^M+1^.

Therefore, a one-dimensional Halton sequence with *k* as the base can be obtained: H_k_(*n*) = η*_k_* (*n*), *n* = 1, 2,…, *N*. A multi-dimensional Halton sequence can be obtained by combining multiple one-dimensional Halton sequences with different bases. The introduction of the Halton sequence in the initialization stage of the grey wolf population ensures a uniform distribution of feasible solutions. However, during the iteration process, GWO relies on the top three solutions to update the positions of the population, which makes it prone to getting trapped in local optima. Therefore, simulated annealing is introduced into GWO to allow α (the lead wolf) to be replaced by a current worse individual with a certain probability. In addition, in order to balance both local and global search capabilities of the algorithm, an incremental strategy is adopted to determine the replacement probability *p* for *α*.

#### 2.3.4 Baseline

K-Nearest Neighbor (KNN) (26) is a non-parametric, supervised learning classifier. The idea behind this method is very simple and intuitive: if a sample belongs to the majority class of its k most similar (i.e., nearest) samples in the feature space, then it also belongs to that class.

Random Forest (RF) (27) algorithm is a popular and powerful supervised machine learning technique that can handle both regression and classification tasks. It creates a forest of decision trees, with the accuracy and reliability of predictions improving as more trees are included in the forest. In regression tasks, the algorithm combines output estimates from multiple trees, while in classification tasks it uses a voting system to determine the class that receives the most votes from all the other trees in the forest.

Decision Tree(DT) (28) is a predictive analytics technique that uses a tree-like graph to predict the value of a target variable based on a set of predictors. It employs divide and conquer problem-solving strategies, starting from the root node with all the data and intelligently splitting it into multiple branches. The goal is to create more homogeneous groups at each child node.

Naive Bayes(NB) (5) algorithm is a classification method that relies on event probability and misclassification loss. Its main advantage is the use of the attribute conditional independence assumption strategy, which helps avoid the issue of combinatorial explosion during posterior probability calculations.

The XGBoost (29) is an advanced modification of the Gradient Boosting technique. It combines predictions from a set of “weak” learners to create a more powerful learner. XGBoost aims to prevent overfitting while optimizing computation. It simplifies objective functions, allowing for the combination of predictive and regularization terms without sacrificing computational speed. In the XGBoost process, the first learner is fitted to the entire input data space. Then, a second model is fitted to these residuals in order to address weaknesses in the initial learner. This fitting process continues iteratively until a stopping criterion is met. The final model is obtained by summing up the predictions from each individual learner.

### 2.4 Experiments and evaluation metrics

In this work, we conducted a systematic analysis of the model’s performance results and presented them using five performance indicators to evaluate the robustness and effectiveness of the outcomes.

#### 2.4.1 Cross validation

**Figure 2:**
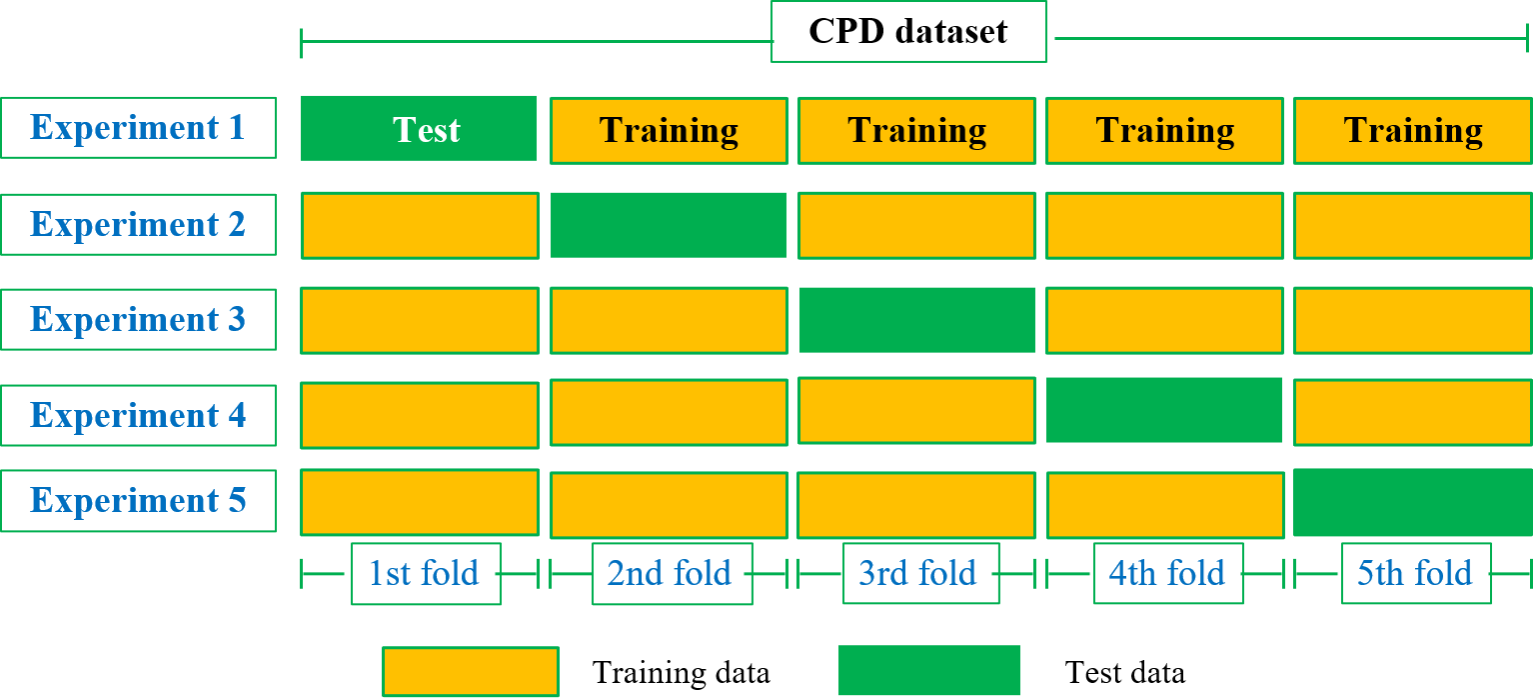
5-fold cross-validation.

This paper utilizes the 5-fold cross-validation approach to create training and test sets. The dataset is initially divided into 5 mutually exclusive subsets (D1-D5) that are equal in size and have a reliable distribution. In each round, one subset is used as the test set while the remaining subsets serve as the training set. The final result is obtained by averaging the results from all five sets. The schematic diagram of the 5-fold cross-validation method is shown in **Figure 2**.

#### 2.4.2 Performance metrics

Confusion matrix is a specific table layout used for visualizing evaluation metrics. Each row in the matrix represents the predicted class of the model, while each column represents the actual class of the samples. It facilitates comparing the predicted results with the actual sample classes to evaluate the performance of the model. The confusion matrix consists of four basic indicators, i.e., TP, FN, FP, and TN. The parameters such as precision, recall, accuracy, F1-score, etc. are calculated as:

• **Precision**: The proportion of correctly predicted positive samples to all predicted positive samples, used to measure the accuracy of the model’s predictions.


(9) $$Precision=\frac{TP}{TP+FP}$$

• **Recall**: The proportion of correctly predicted positive samples to all positive samples.


(10) $$Recall=\frac{TP}{TP+FN}$$

• **Accuracy**: The proportion of correctly predicted samples in the prediction model to the total number of observations.


(11) $$Accuracy=\frac{TN+TP}{TN+TP+FN+FP}$$

• **F1-score**: This metric combines the output results of Precision and Recall, which is the average harmonic value of both. The F1-Score ranges from 0 to 1, where 1 represents the best output result of the model, while 0 indicates otherwise.


(12) $$F1-score=\frac{2*\left(Precision*Recall\right)}{Precision+Recall}$$

• **AUC**: The AUC value is calculated by measuring the area under the ROC curve. The ROC curve compares the performance of classifiers across different discrimination thresholds in terms of true positive rate and false positive rate. This makes the AUC value a reliable measure for comparing classification algorithms. Equation 13 is used to calculate the AUC based on TP, FN, FP, and TN values.


(13) $$AUC=\frac{1}{2}\left(\frac{TP}{TP+FN}+\frac{TN}{TN+FP}\right)$$

## 3 Results

### 3.1 Data sources and descriptions

This study utilized the electronic medical record system of a reproductive center to obtain comprehensive patient information for research purposes. The dataset included records of 840 patients who underwent ART. **Table 1** provides details about the 19 feature variables and 1 categorical label. The variables in **Table 1** are classified into two categories: 6 qualitative variables and 13 quantitative variables. The label variable, CP (clinical pregnancy), had a value of 0 indicating no pregnancy (including 90 cases of biochemical pregnancy), and a value of 1 indicating clinical pregnancy.

**Table 1:** Description of features.

No.	Type	Feature name	Description	Feature value (Mean value) (Range)
1	Categorical	Scheme	Scheme	1: long protocol 2: GnRH protocol 3: short protocol 4: natural cycles 5: antagonist protocol
2		UFP	Ultimate Fertility Program	0: ICSI 1: IVF
3		HSG	Hysterosalpingography	0: abnormal 1: normal 2: unknown
4		Gravidity	Gravidity	0: primary infertility 1: secondary infertility
5		IF	Infertility factor	0: men 1: woman 2: both 3: unknown
6		EQ	Embryo quality on embryo transfer day	0: embryo 1: blastocyst
7	Numerical	Age	Age of woman	32.25 (4.44) (22-49)
8		BMI	Body mass index	23.34 (3.32) (16.4-37.2)
9		Years	Years of infertility	4.04 (2.80) (2-17)
10		tdose	Total Gonadotrophin dose	3144.51 (1285.60) (75-12275)
11		days	Duration of Stimulation	10.64 (2.57) (9-26)
12		Retries to ART	Retries to assisted reproductive technology	1.81 (1.39) (1-14)
13		EM TNK	Endometrium thickness (mm) on HCG day	10.74 (2.84) (3-12)
14		etwo	Oestrogen concentration at the HCG injection	2248.39 (1510.55) (0.64-14030)
15		mtwo	Mature oocyte count	8.25 (4.77) (1-33)
16		ETD	Embryo transfer day	4.58 (0.98) (3-6)
17		No. of ET	Number of transfer embryos	1.57 (0.50) (1-3)
18		SC	Total sperm count	49.39 (36.78) (1-212)
19		SM	Total sperm motility	39.15 (17.74) (0-91)
20	Label	CP	Clinical pregnancy	0: no pregnancy 1: clinical pregnancy

### 3.2 Description of statistics

**Figure 3:**
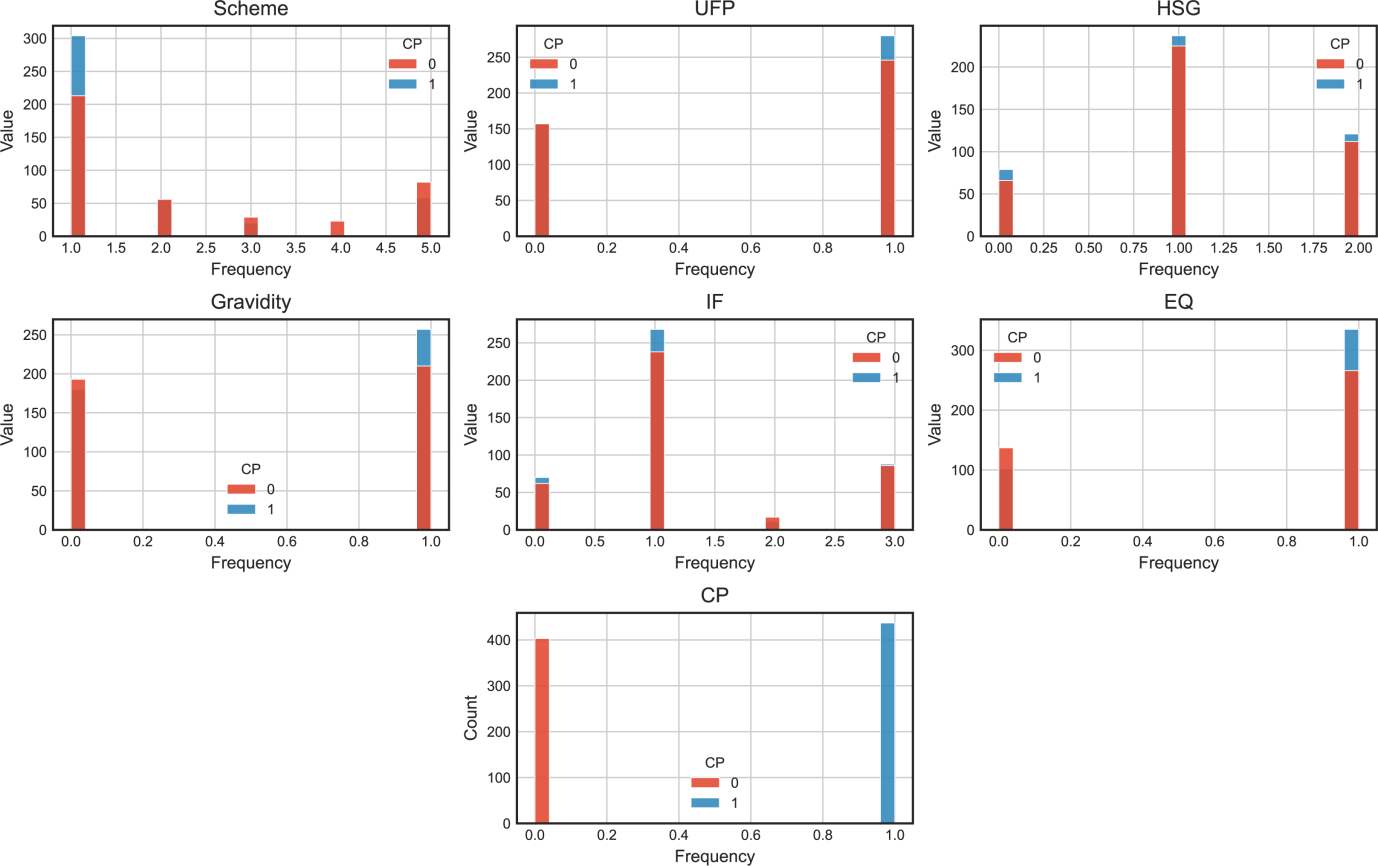
The frequency distribution histogram of CP Dataset with category features.

The frequency distribution histogram of CP Dataset with category features was shown in **Figure 3**. The label (CP) represents whether a clinical pregnancy occurred. The blue portion represents patients with successful clinical pregnancies, totaling 437 cases and accounting for 52.02% of the dataset. The orange portion represents patients without clinical pregnancies, including biochemical pregnancies, totaling 403 cases and accounting for 47.98% of the dataset. The label feature exhibits a small difference and is close to a balanced state, which facilitates subsequent analysis.

After analyzing the quantity and proportion of label features, let’s examine the data information separately for numerical features and categorical features. Each categorical feature consists of 840 samples. To provide a visual representation, we have created histograms for 6 categorical feature variables, as depicted in **Figure 2**. Among the different protocols, the long protocol has the highest number of samples, with 517 cases and a clinical pregnancy rate of 58.80%. The GnRH protocol has 111 samples, with a clinical pregnancy rate of 49.55%. The short protocol has 49 samples, with a clinical pregnancy rate of 40.82%. The natural cycle has 23 samples, with no clinical pregnancies. Finally, the antagonist protocol has 140 samples, with a clinical pregnancy rate of 41.43%. The long protocol exhibits the highest clinical pregnancy rate, while the natural cycle has the lowest. Regarding the final assisted reproductive plan, there are 314 samples with ICSI fertilization, resulting in a clinical pregnancy rate of 50.00%. There are 526 samples with IVF fertilization, resulting in a clinical pregnancy rate of 39.54%. For fresh transfer cycles, the clinical pregnancy rate is relatively higher for ICSI fertilization compared to conventional fertilization. In terms of uterine tubal patency testing, there are 145 samples with abnormal findings, resulting in a clinical pregnancy rate of 54.48%. There are 462 samples with normal findings, resulting in a clinical pregnancy rate of 51.30%. Finally, there are 233 samples with unknown fallopian tube patency, resulting in a clinical pregnancy rate of 51.93%. The status of the fallopian tubes appears to have little impact on clinical pregnancy outcomes in fresh transfer cycles, as the embryos are directly transferred to the uterine cavity. Among different infertility types, there are 373 samples of primary infertility, resulting in a clinical pregnancy rate of 48.26%. There are 467 samples of secondary infertility, resulting in a clinical pregnancy rate of 55.03%. In terms of infertility factors, there are 132 samples with male factors, resulting in a clinical pregnancy rate of 53.03%. There are 506 samples with female factors, resulting in a clinical pregnancy rate of 52.96%. There are 28 samples with both male and female factors, resulting in a clinical pregnancy rate of 39.29%. There are 174 samples with unknown causes, resulting in a clinical pregnancy rate of 50.57%. Lastly, in terms of embryo quality, there are 239 samples of transferred embryos, resulting in a clinical pregnancy rate of 42.68%. There are 601 samples of transferred blastocysts, resulting in a clinical pregnancy rate of 55.74%. It is evident that the success rate of blastocysts in fresh transfers is higher than that of embryos.

**Figure 4:**
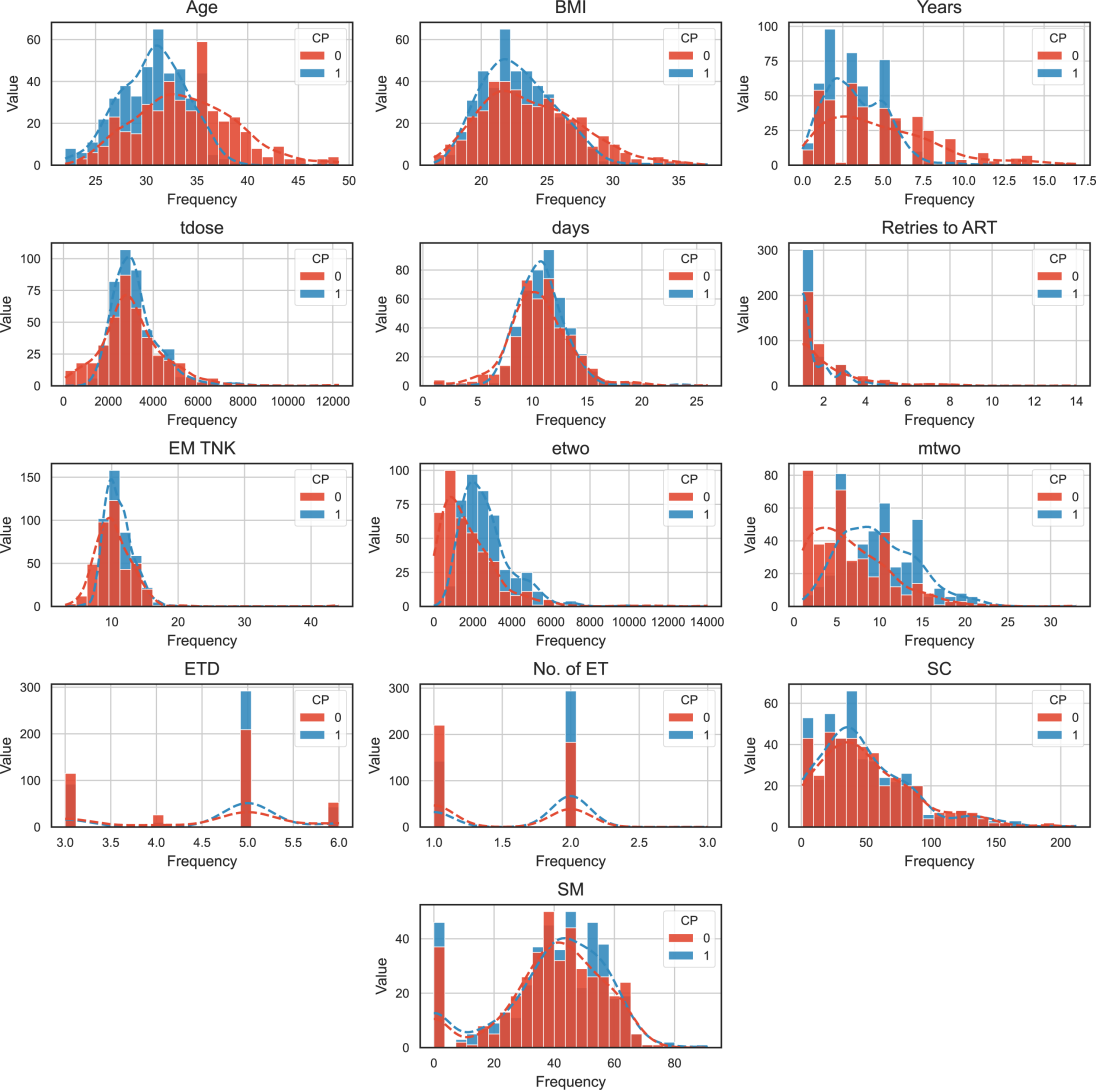
The frequency distribution histogram of CP Dataset with numeric features.

Based on the distribution of 13 numerical feature variables plotted based on different label states, we can observe that the distribution of infertile patients (represented by the red curve) is consistent with the overall feature distribution (represented by the blue curve). This implies that it is difficult to determine which feature is closely related to clinical pregnancy through intuitive inspection alone. Let’s examine the descriptive statistical information for each numerical variable (**Figure 4**):

- **Age**: The minimum and maximum values are 22 and 49, respectively, covering the entire age span of IVF patients. The average age of clinically pregnant patients is 30.65, indicating that clinical pregnancy becomes more difficult with increasing age.
- **BMI**: The minimum and maximum values are 16.4 and 37.2. The average BMI of clinically pregnant patients is 22.89. Being underweight or obese is unfavorable for embryo implantation.
- **Years (duration of infertility)**: The range is from 0 to 17 years. The average duration of infertility for clinically pregnant and non-pregnant patients is 3.29 and 4.84, respectively. The longer the duration of infertility, the more difficult it becomes to conceive.
- **Tdose (total dose of Gn)**: The minimum and maximum values are 75 and 12275, respectively. The average values for clinically pregnant and non-pregnant patients are 3194.75 and 3090.03, respectively, with little difference.
- **Days (total number of Gn days)**: The minimum and maximum values are 1 and 14.
- **Retries to ART (total number of treatment cycles):** The minimum and maximum values are 1 and 14.
- **EM TNK (HCG endometrial thickness)**: The minimum and maximum values are 3 and 44.2. The average HCG endometrial thickness for clinically pregnant and nonpregnant patients is 10.42 and 11.03, respectively, indicating that endometrial thickness has little impact on embryo implantation.
- **etwo (oestrogen concentration at the HCG injection)**: The minimum and maximum values are 0.64 and 12226.3. The average values of etwo for clinically pregnant patients is 2672.7, while for non-pregnant patients it is 1788.29. Higher values of etwo are beneficial for embryo implantation.
- **mtwo (number of mature eggs)**: The minimum and maximum values are 1 and 33. The average numbers of mature eggs for clinically pregnant and non-pregnant patients are 9.83 and 6.53, respectively. Having more mature eggs is beneficial for the clinical pregnancy outcome.
- **ETD (embryo transfer day)**: The ETD for clinical pregnancy and non-pregnant patients are on the 3rd, 4th, 5th, and 6th day after oocyte retrieval. The average days of embryo transfer after oocyte retrieval are 4.50 and 4.65 for clinically pregnant and non-pregnant patients, respectively. The maximum number of patients who had embryo transfer on the 5th day after oocyte retrieval is 501.
- **ET (number of transferred embryos)**: The number of ET is 1, 2, and 3 (with only 1 case meeting the criteria for multiple embryo transfer). There were 362 cases of single embryo transfer and 477 cases of double embryo transfer.
- **SC (sperm count)**: The minimum and maximum values are 1 (azoospermia and occasional patients) and 212. The average sperm counts for male partners of clinically pregnant and non-pregnant patients are 48.44 and 50.41, respectively.
- **SM (sperm motility)**: The minimum and maximum values are 0 (azoospermia and occasional patients) and 91. The average sperm motilities for male partners of clinically pregnant and non-pregnant patients are 39.16 and 39.13, respectively. From these statistics, we can see that the male semen condition (SC and SM) has little impact on clinical pregnancy outcomes, while factors such as age, BMI, Years, etwo, mtwo, and ETD may play a role in determining the likelihood of clinical pregnancy.

### 3.3 Correlation

**Figure 5:**
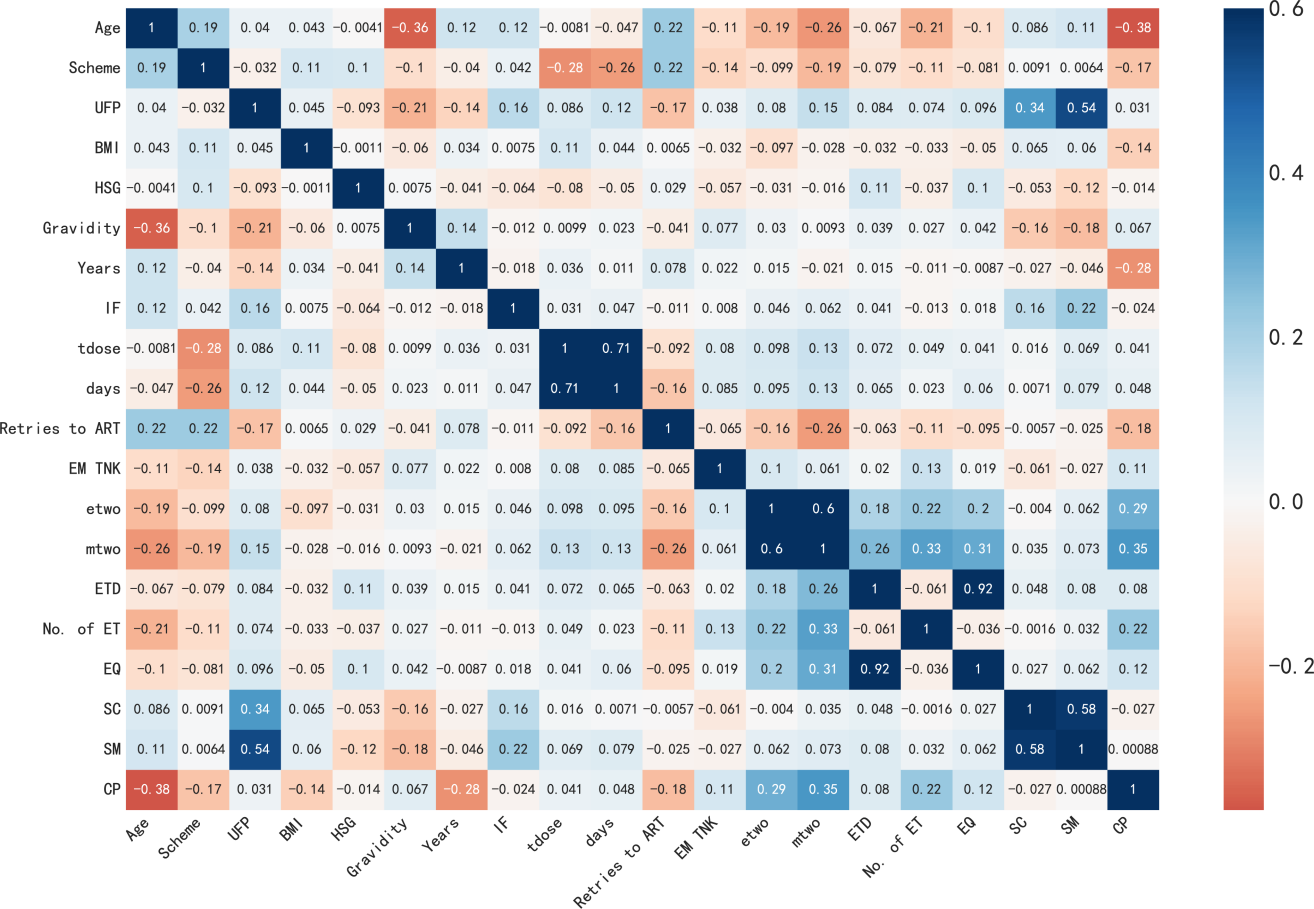
The correlation matrix of features.

The correlation between features plays a vital role in the feature selection stage within an academic setting. To measure this correlation, we utilize the Pearson correlation coefficient. Positive values denote a positive correlation, whereas negative values represent a negative correlation. In **Figure 3**, the color depth on the right scale reflects different correlation coefficients, with darker colors indicating a stronger correlation and vice versa. The dataset exhibits consistent Pearson correlation coefficients among its features, except for Age, Scheme, BMI, HSG, Years, IF, Retries to ART, and SC, which demonstrate negative correlations. On the other hand, the remaining 10 attributes display positive correlations with the target variable. The listed features have independent effects on the label column, as can be seen from **Figure 5**, because the correlation coefficients of each feature are less than 0.3. However, we can see that feature SM is correlated with SC and UFP, and ETD is also significantly correlated with EQ. To avoid the influence of multicollinearity on the prediction model, we excluded the variables SC, UFP, and ETD in our experiment.

### 3.4 Models’ comparison

In this section, we trained six algorithms using a 5-fold cross-validation method and validated the proposed framework using the CPD dataset. **Table 2** presents the average performance of these algorithms on four metrics including accuracy, precision, recall and F1-score. Additionally, **Table 3** shows the confusion matrices of these six algorithms, detailing the percentages of TP, FP, TN, and FN cases in their predicted results.

**Table 2:** The results of six algorithms.

Alorithm	Accuracy	Precision	Recall	F1-score
KNN	0.7024	0.6633	0.7927	0.7009
Naïve Bayes	0.7976	0.7857	0.8049	0.7976
Decision Tree	0.8393	0.8395	0.8293	0.8391
Random Forest	0.8690	0.8750	0.8537	0.8689
XGBoost	0.8750	0.8861	0.8537	0.8696
LightGBM	0.9048	0.9231	0.8780	0.9000

**Table 3:** The confusion matrix of six algorithms.

Algorithm	Confusion matrix			Description
**KNN**	Total Population **168**	Predicted **No** Pregnancy	Predicted Pregnancy	**TN**: In 53 samples, the CP outcome was successfully predicted, and the anticipated results and actual sample were congruent without pregnancy. **TP :** In 65 samples, the CP outcome was successfully predicted, and the anticipated results and actual sample were congruent with CP. **FP**: In 33 samples, the CP outcome was incorrectly predicted, anticipated results indicated CP and actual sample without pregnancy. **FN**: In 17 samples, the CP outcome was incorrectly predicted, anticipated results without pregnancy and actual samples indicated CP.
	Actual **No** Pregnancy **86**	True Negative(TN) **53**	False Positive(FP) **33**	
	Actual Pregnancy **82**	False Negative(FN) **17**	True Positive(TP) **65**	
**NB**	Total Population **168**	Predicted **No** Pregnancy	Predicted Pregnancy	**TN**: In 68 samples, the CP outcome was successfully predicted, and the anticipated results and actual sample were congruent without pregnancy. **TP :** In 66 samples, the CP outcome was successfully predicted, and the anticipated results and actual sample were congruent with CP. **FP**: In 18 samples, the CP outcome was incorrectly predicted, anticipated results indicated CP and actual sample without pregnancy. **FN**: In 16 samples, the CP outcome was incorrectly predicted, anticipated results without pregnancy and actual samples indicated CP.
	Actual **No** Pregnancy **86**	True Negative(TN) **68**	False Positive(FP) **18**	
	Actual Pregnancy **82**	False Negative(FN) **16**	True Positive(TP) **66**	
**DT**	Total Population **168**	Predicted **No** Pregnancy	Predicted Pregnancy	**TN**: In 73 samples, the CP outcome was successfully predicted, and the anticipated results and actual sample were congruent without pregnancy. **TP :** In 68 samples, the CP outcome was successfully predicted, and the anticipated results and actual sample were congruent with CP. **FP**: In 13 samples, the CP outcome was incorrectly predicted, anticipated results indicated CP and actual sample without pregnancy. **FN**: In 14 samples, the CP outcome was incorrectly predicted, anticipated results without pregnancy and actual samples indicated CP.
	Actual **No** Pregnancy **86**	True Negative(TN) **73**	False Positive(FP) **13**	
	Actual Pregnancy **82**	False Negative(FN) **14**	True Positive(TP) **68**	
**RF**	Total Population **168**	Predicted **No** Pregnancy	Predicted Pregnancy	**TN**: In 76 samples, the CP outcome was successfully predicted, and the anticipated results and actual sample were congruent without pregnancy. **TP :** In 70 samples, the CP outcome was successfully predicted, and the anticipated results and actual sample were congruent with CP. **FP**: In 10 samples, the CP outcome was incorrectly predicted, anticipated results indicated CP and actual sample without pregnancy. **FN**: In 12 samples, the CP outcome was incorrectly predicted, anticipated results without pregnancy and actual samples indicated CP.
	Actual **No** Pregnancy **86**	True Negative(TN) **76**	False Positive(FP) **10**	
	Actual Pregnancy **82**	False Negative(FN) **12**	True Positive(TP) **70**	
**XGBoost**	Total Population **168**	Predicted **No** Pregnancy	Predicted Pregnancy	**TN**: In 77 samples, the CP outcome was successfully predicted, and the anticipated results and actual sample were congruent without pregnancy. **TP :** In 70 samples, the CP outcome was successfully predicted, and the anticipated results and actual sample were congruent with CP. **FP**: In 9 samples, the CP outcome was incorrectly predicted, anticipated results indicated CP and actual sample without pregnancy. **FN**: In 12 samples, the CP outcome was incorrectly predicted, anticipated results without pregnancy and actual samples indicated CP.
	Actual **No** Pregnancy **86**	True Negative(TN) **77**	False Positive(FP) **9**	
	Actual Pregnancy **82**	False Negative(FN) **12**	True Positive(TP) **70**	
**LightGBM**	Total Population **168**	Predicted **No** Pregnancy	Predicted Pregnancy	**TN**: In 80 samples, the CP outcome was successfully predicted, and the anticipated results and actual sample were congruent without pregnancy. **TP :** In 72 samples, the CP outcome was successfully predicted, and the anticipated results and actual sample were congruent with CP. **FP**: In 6 samples, the CP outcome was incorrectly predicted, anticipated results indicated CP and actual sample without pregnancy. **FN**: In 10 samples, the CP outcome was incorrectly predicted, anticipated results without pregnancy and actual samples indicated CP.
	Actual **No** Pregnancy **86**	True Negative(TN) **80**	False Positive(FP) **6**	
	Actual Pregnancy **82**	False Negative(FN) **10**	True Positive(TP) **72**	

Accuracy is the key indicator used to evaluate model performance, with LightGBM achieving an impressive accuracy of 90.48%. Similarly, XGBoost, RF, and DT models attain respective accuracies of 89.45%, 88.90%, and 83.93%. Conversely, NB and KNN models exhibit accuracies lower than 80.00%, specifically 79.76% and 72.24% respectively. Notably, precision and recall, two evaluation metrics, often demonstrate an inverse correlation trend wherein higher precision corresponds to lower recall, and vice versa. In this particular experiment, except for the XGBoost, LightGBM, RF, and DT models, the precision of KNN and NB models falls below their recall values. In addition, whereas LightGBM showcases outstanding performance with an F1-Score of 90.00%.

**Figure 6:**
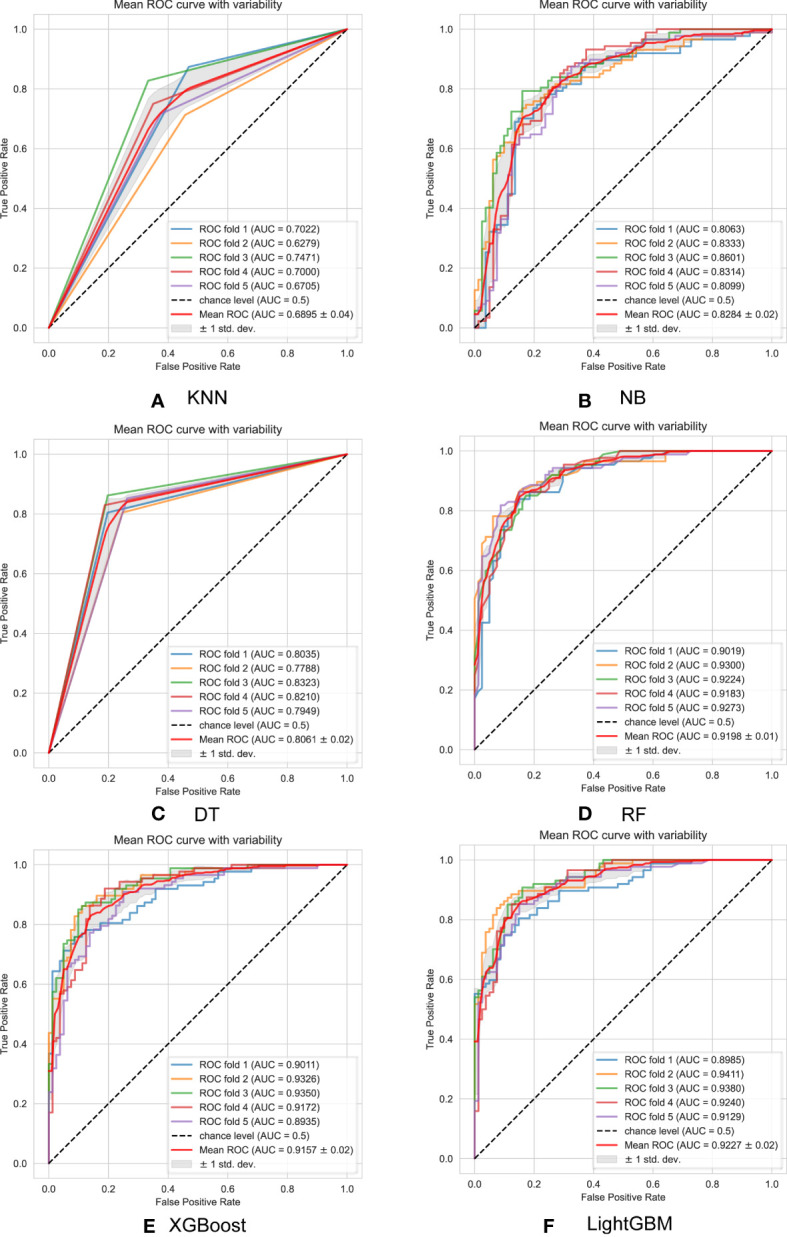
ROC curves are used to evaluate the performance of six machine learning models with 5-fold cross-validation: **(A)** KNN model; **(B)** NB model; **(C)** DT model; **(D)** RF model; **(E)** XGBoost model; **(F)** LightGBM model.

The evaluation of model performance utilizes the Receiver Operating Characteristic (ROC) curve and calculates the Area under the ROC (AUC) as descriptors in **Figure 6**. A higher AUC value indicates a stronger generalization ability of the model, as seen by the ROC curve approaching the top left corner of the graph. The mean Receiver Operating Characteristic (ROC) for the trained models, namely KNN, NB, DT, RF, XGBoost and LightGBM, using a five-fold cross-validation method, are as follows: 68.95% ± 0.04, 82.84% ± 0.02, 80.61% ± 0.02, 91.57% ± 0.02, 92.10% ± 0.01, and 92.27% ± 0.02, respectively. The RF, XGBoost, and LightGBM perform well, with AUC values all above 90%. Among them, LightGBM achieves the highest AUC value of 92.27 ± 0.02%, However, the KNN and NB models perform poorly, achieving AUC values of only 70.45% and 79.78% respectively. In other words,the proposed model significantly outperformed by at least 23.32% compared to these lowest performing models.

When considering all evaluation metrics, the LightGBM algorithm demonstrates significant advantages in predicting successful pregnancy during *in vitro* fertilization treatment cycles, closely followed by XGBoost. Furthermore, the comparison highlights the subpar performance of both the k-nearest neighbors and naive bayes algorithms.

**Figure 7:**
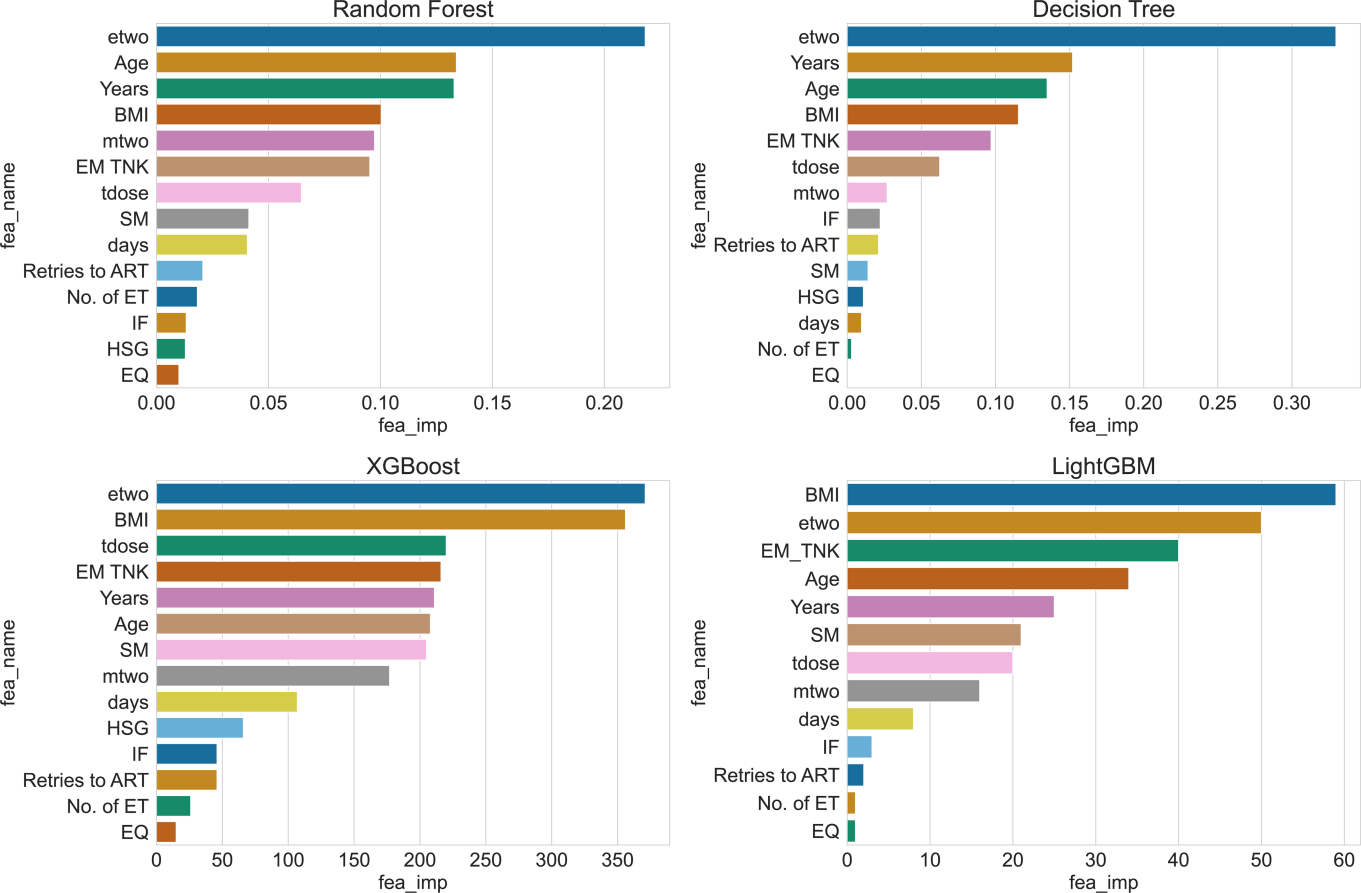
Ranking of feature importance for 4 algorithms.

The impact of the 14 features in the IVF dataset on the prediction results is unique for each feature. Different models exhibit preferences for specific features, resulting in varying scores for these features. However, the KNN and NB algorithms do not provide internal feature importance evaluation. Therefore, we present the feature importance rankings of RT, DT, XGBoost and LightGBM in **Figure 7**.

By examining the feature rankings in **Table 4**, it becomes evident that the concentration of estrogen at the time of HCG injection (etwo) plays a crucial role in predicting clinical pregnancy. EM TNK, Years, and BMI consistently appear among the top five important features in all four algorithms. These factors are influential and should not be disregarded during prediction. Moreover, the table reveals that Decision Tree and Xgboost algorithms generate similar feature importance rankings since both algorithms construct the same tree structure during training.

**Table 4:** Features ranking of 4 algorithms.

Ranking	Random Forest	Decision Tree	XGBoost	LightGBM
1	etwo	etwo	etwo	BMI
2	Years	Years	BMI	etwo
3	Age	Age	tdose	EM TNK
4	BMI	BMI	EM TNK	Age
5	EM TNK	EM TNK	Years	Years

Overall, we used a comparative approach to demonstrate the prediction results of the LightGBM algorithm and selected benchmark algorithms on the processed dataset. Based on the information from the confusion matrix showing four different prediction outcomes, we calculated accuracy, precision, recall, and F1 score. We also plotted receiver operating characteristic (ROC) curves for each algorithm using 5-fold cross-validation. The experimental results showed that our proposed algorithm performed exceptionally well in all evaluation metrics, demonstrating significant advantages in predicting clinical pregnancy based on the GWO-LightGBM algorithm framework. Additionally, we estimated feature importance and correlation scores for these four algorithms, providing valuable insights for future algorithm optimization.

## 4 Discussion

Traditional statistical methods are not suitable for establishing prediction models for the outcome of IVF-ET treatment due to the influence of various factors and the complex interactions among them, which violate the assumption of independence between variables. Machine learning methods, in contrast to traditional statistical methods, have the ability to effectively model complex systems by considering the intricate relationships and associations among variables. This approach affords the opportunity to generate unbiased and robust models for future predictions, facilitating synergy between various parameters that may not have direct connections to the outcomes (30). On the other hand, machine learning methods offer potential for improving the pregnancy rate after assisted reproductive technology (ART) treatment. However, despite numerous reports on prediction models for IVF-ET treatment outcomes both domestically and in- ternationally, these models exhibit limitations, such as low prediction accuracy, limited sample sources (mostly relying on national large sample databases), and a lack of baseline characteristics of the study population.

It is widely recognized that improving clinical pregnancy rates has always been a key focus and challenge in the application and promotion of assisted reproductive technology. Through the utilization of machine learning to establish predictive models, clinical doctors can make adjustments to relevant adverse factors during the *in vitro* fertilization embryo transfer (IVF-ET) process for infertile couples, providing personalized consultations to improve treatment outcomes (31, 32). Various studies in the literature aim to predict clinical pregnancy outcomes. These studies utilize a range of variables, data processing methods, and machine learning techniques, hindering direct comparisons. Among them, researchers have primarily focused on technological innovations in data processing, particularly in the area of feature selection, as well as advancements in predictive algorithms, during the application of machine learning for predicting clinical pregnancy outcomes.

In studies predicting clinical pregnancy outcomes after ART, a minimum of 4 features was reported (19), and the maximum number was 82 (33). Based on statistical data, among the 25 studies, the variable analysis in 10 studies involved the use of embryological, clinical, and demographic data in the IVF dataset, while only five studies incorporated sperm parameters into these variables. In the current research, most studies only utilized day 2-3 embryo transfers, while only one study included both day 5 blastocyst transfers and day 3 embryo transfers (34). In comparison to embryos, blastocysts demonstrate a higher degree of synchronization with the uterine lining. Furthermore, transfer at the blastocyst stage creates a more physiologically natural environment and possesses a greater capacity for implantation. Consequently, there has been an increasing number of patients undergoing blastocyst transfers. Including the day 5 transfer variable has a significant impact on the results, and assists in attaining more realistic and feasible outcomes (35).

This study utilized five machine learning methods and concluded that the concentration of estradiol, years of infertility, BMI, and endometrium thickness on HCG Day were the top four most important variables. Concentration of estradiol, as an indicator of ovarian reserve, was confirmed as the primary predictive variable, which is consistent with findings from previous research (36–39). The years of infertility, as the second important variable, aligns with the findings of Linda’s study (40). Moreover, BMI, as a measure of body health, has the potential to disrupt hormonal balance, decrease fertilization and oocyte maturation rates, and negatively impact oocyte or embryo quality, consequently influencing pregnancy outcomes (41, 42). The incorporation of BMI as a significant predictive indicator highlights a specific issue in present-day infertility. Bearing in mind the vital role of the uterine endometrium in embryo implantation, its inclusion as a variable signifies an essential factor influencing clinical pregnancy outcomes.

In 2013, Güvenir et al. (43) conducted a study using the basic information and IVF cycle data of 1456 infertile couples from a local assisted reproductive institution. They utilized three different algorithms, namely the SERA algorithm, Naïve Bayes, and Random Forest, to develop a predictive model for successful pregnancy outcomes. These algorithms were employed to analyze a total of 64 independent predictive factors. The AUC values for the three algorithmic models were determined to be 0.833, 0.794, and 0.769, respectively. It’s AUC of the model proposed in this study is significantly higher than the findings of previous reports, but it was much lower than the AUC value of 0.9227 predicted by the LightGBM model in this study. Untill to 2020, Hassan et al. (44) recruited a sample of 1048 patients and utilized five algorithms, including Support Vector Machines (SVM) and random forest, to develop a predictive model for pregnancy. The study employed two methods, namely feature selection and non-feature selection, to construct predictive models for these algorithms. The findings of the study suggest that the performance of the predictive models significantly improved after applying feature selection. Moreover, the study identified the SVM model, following feature selection, as the most effective with an impressive AUC of 0.995. which is higher than our study. But in contrast, our study employed a more extensive range of evaluations, utilizing machine learning model performance as the basis. A comprehensive comparative analysis was conducted to assess the applicability and robustness of the models.

Generally, machine learning models excel in predicting the clinical pregnancy outcome of IVF-ET treatment. The significant variables identified by our proposed LightGBM algorithm align closely with the existing literature. Moreover, by comparing it with other standard algorithms, we have confirmed the effectiveness and accuracy of our proposed algorithm.

## 5 Conclusion

In this study, we collected samples from patients in our reproductive center and employed six representative algorithms, considering comprehensive feature values, to examine the use of machine learning algorithms in predicting the clinical pregnancy outcome of IVF-ET. Our findings suggest that the LightGBM model demonstrates superior predictive capabilities and classification accuracy, making it an ideal model for forecasting clinical pregnancy outcomes in future assisted reproductive technologies. Furthermore, we discovered that the concentration of estradiol, years of infertility, BMI, and endometrium thickness on HCG Day are the four most significant variables. These results will enhance fertility specialists’ ability to predict IVF cycle outcomes, provide consultative guidance to patients, and further determine the importance of each IVF variable in successful treatment, thus prompting the development of novel strategies to optimize these variables.

## Data availability statement

Data is available upon reasonable request. Requests to access these datasets should be directed to yangj@sxufe.edu.cn.

## Ethics statement

The studies involving humans were approved by Ethics Committee of Shanxi Medical University. The studies were conducted in accordance with the local legislation and institutional requirements. Written informed consent for participation was not required from the participants or the participants’ legal guardians/next of kin in accordance with the national legislation and institutional requirements.

## Author contributions

LL: Formal analysis, Investigation, Methodology, Writing – original draft, Writing – review & editing. XC: Conceptualization, Funding acquisition, Writing – review & editing. JY: Conceptualization, Formal analysis, Funding acquisition, Investigation, Methodology, Writing – original draft, Writing – review & editing. XW: Supervision, Writing – review & editing. GZ: Supervision, Writing – review & editing.
